# Understanding of cervical cancer, acceptability of HPV self-collection, and prevalence of HPV in a semi-urban setting in Bangladesh

**DOI:** 10.1371/journal.pgph.0003157

**Published:** 2024-04-24

**Authors:** Lilah Khoja, Yuting Wang, Syed Emdadul Haque, Habibul Ahsan, Tariqul Islam, Saif Ullah Munshi, A. K. M. Rabiul Hasan, Md. Tariqul Islam, Alaya Begum Jharna, Celeste Leigh Pearce

**Affiliations:** 1 Department of Epidemiology, University of Michigan School of Public Health, Ann Arbor, Michigan, United States of America; 2 UChicago Research Bangladesh, Dhaka, Bangladesh; 3 Institute for Population and Precision Health, University of Chicago, Chicago, Illinois, United States of America; 4 Center for Multidisciplinary Research, Gono Bishwabidyalay, Dhaka, Bangladesh; 5 Department of Virology, Bangabandhu Sheikh Mujib Medical University, Dhaka, Bangladesh; 6 URB Araihazar Field Office, Narayanganj, Bangladesh; African Union, ETHIOPIA

## Abstract

*Human Papillomavirus* (HPV) self-sampling has been implemented successfully as an alternative to traditional forms of cervical cancer screening in low-resource settings. Through Bangladesh’s current national cervical cancer screening program, only about 10% of the at-risk population is reached. Thus, Bangladesh is an ideal setting to consider HPV self-sampling to improve cervical cancer prevention efforts. However, the feasibility and acceptability of HPV self-sampling has not been evaluated in Bangladesh. We aimed to understand levels of HPV and cervical cancer knowledge and to evaluate the feasibility and acceptability of HPV self-sampling for cervical cancer screening in a semi-urban Bangladeshi community. Participants were recruited from a local clinic; 164 women completed a cross-sectional questionnaire about attitudes towards screening, and cervical cancer and HPV risk factor knowledge, and provided self-collected cervical samples for high-risk HPV testing. Of the participants, 4.3% tested positive for high-risk HPV and were referred for appropriate follow-up care. Nearly all participants had heard of cervical cancer, though specific knowledge was quite low. Self-sampling for high-risk HPV testing had high rates of acceptability, high rates of convenience, and very little discomfort and embarrassment reported in this study population, making implementing HPV self-sampling as a form of cervical cancer screening in Bangladesh appear feasible.

## Introduction

Through a combination of vaccination and appropriately timed screening, cervical cancer is preventable. Despite this, cervical cancer is the most diagnosed gynecologic cancer amongst women worldwide, with more than 600,000 new cases and 340,000 deaths worldwide in 2020 [1]. Risk factors for cervical cancer include high-risk *Human papillomavirus* (HPV) infection, tobacco use, contraceptive use, and increased parity [2–7].

The introduction of HPV self-sampling as an alternative to traditional screening methods, such as a Pap test, has shown great success as both a primary method of screening and as a method to reach under-screened populations [8, 9]. HPV self-sampling is less resource-intensive than cytology and has been evaluated for use in limited-resource settings [10–17]. Self-sampling has been found to have high levels of acceptability in low- and middle-income countries, including in Muslim-majority communities [13, 14, 18, 19], such as Cameroon [17], Guatemala [16], India [11], Nicaragua [11, 12], Uganda [11, 20], Liberia [19], Senegal [14], Malaysia [18, 21], and Malawi [22], among others.

In Bangladesh, cervical cancer is the second most diagnosed cancer amongst women, with 8,000 incident cases annually and more than 5,000 deaths [23]. In 2017, the Bangladesh Cancer Control Strategy identified cervical cancer as an area of concern [24]; 80% of cases are diagnosed at Stage III or IV, and treatment availability at those stages is scarce resulting in high cervical cancer mortality [25]. Visual inspection with acetic acid (VIA) is the primary method of cervical cancer screening in Bangladesh [26], but less than 10% of the at-risk population is reached [27]. The low uptake of cervical cancer screening in Bangladesh is due to lack of access to screening [27] and lack of knowledge of cervical cancer and screening methods [28].

Currently, HPV self-sampling for cervical cancer screening is not available in Bangladesh. However, given the low screening uptake and the utility of HPV self-collection in low-resource settings, Bangladesh is an ideal setting to consider HPV self-collection to improve cervical cancer prevention efforts. However, to our knowledge, feasibility and acceptability of HPV self-sampling has not been evaluated in Bangladesh. Therefore, through this study, we aimed to understand knowledge of HPV and cervical cancer and to evaluate the feasibility and acceptability of HPV self-sampling for cervical cancer screening in a semi-urban Bangladeshi community.

## Methods

This cross-sectional study was developed by the UChicago Research Bangladesh (URB) and the University of Michigan, Department of Epidemiology as part of our ongoing collaborative research partnership. IRB approval was sought and received from the Bangladesh Medical Research Council (BMRC) and Bangabandhu Sheikh Mujib Medical University (BSMMU). The University of Michigan (UM) Health Sciences and Behavioral Subjects (HSBS) Institutional Review Board assigned the study a “not regulated” designation as UM investigators received only coded data for analysis. Written informed consent was obtained from all participants. All participants received an incentive of 700 taka, or approximately 7 US dollars.

### Study population

Participants were selected randomly from eligible women who utilized the URB primary health care center, located in Araihazar Upazila, Bangladesh between June 2021 –January 2022. Random selection was achieved by asking every other eligible patient to participate in the study. Women aged between 30 and 65 years, with no history of prior abnormal cervical cancer screening or treatment/procedures on their cervix and with no history of cervical cancer were eligible to participate. Women with a history of any cancer, with a history of chronic kidney or liver disease, and those who were critically ill were ineligible to participate. Pregnant women were also excluded from participating. A total of 200 women were invited to participate; 164 women enrolled in the study resulting in an 82% participation rate. After initial eligibility was established based on age, women were invited to a private area to continue the rest of the eligibility screening. Once eligibility was confirmed, women were consented into the study.

### Biospecimen collection

Once a participant consented, they were asked to complete the biospecimen self-collection. Participants were provided with the HerSwab (Eve Medical Inc, Canada), an HPV self-sampling collection kit, along with an instructional flyer featuring both infographic and text instructions. The infographic included how to open the kit, position oneself, properly use the device, and store it after use. The text instructions were in Bangla. Participants were additionally provided with direction by a nurse trained by the gynecologist.

Participants were directed to the bathroom to collect the sample. After collection, the device was placed into a plastic bag and returned to the study team nurse. A subset (n = 30) of participants who self-sampled were randomly selected to have a trained nurse collect their sample, allowing us to assess the concordance in self- and provider- sample collection.

Biospecimens were tested for high-risk HPV infection using the digene HC2 HPV DNA Test. Test results were categorized as positive for high-risk HPV infection, negative for high-risk HPV infection, or borderline (uninterpretable). Women who tested positive were advised to seek further testing at the National Cancer Center housed at BSMMU. The URB team communicated with BSMMU to support participants who tested positive. Those with a borderline test result were advised to seek follow-up testing in two years.

### Data collection

Participants were asked to self-complete a brief questionnaire in Bangla. Those who were unable to self-complete had the assistance of trained staff. The questionnaire was divided into four sections: demographics, lifestyle, health, and attitudes towards cervical cancer screening practices, knowledge of HPV/cervical cancer, and attitudes towards the HPV self-collection test [16, 29].

### Variable categorization and measures

Demographic characteristics included age, marital status, education, and occupation. Age was collected as a continuous variable and was categorized into three groups (30–39, 40–49, and 50–65). Marital status was categorized as single, married, divorced, or separated, and widowed. Education categories followed the structure of the Bangladeshi educational system: no education (0 years of schooling), primary education (1–5 years), secondary school (6–10 years), higher secondary school (11–12 years), graduate (13–16 years), and post-graduate (17 years). The higher secondary education is equivalent to completion of a U.S. high school education. For occupation, though participants were given a range of options, only the selected three categories are presented. One participant selected “other” and had a write-in answer that allowed for them to recategorized as “service worker”, an existing occupation category.

Lifestyle factors included gravidity, number of living children, and smoking status. Number of living children was collected as a categorical variable (never been pregnant, one time, two times, three times, and four or more times). Parity was collected similarly. Smoking status was also collected as a categorical variable, with the following options: never smoker, used to smoke occasionally but quit, used to smoke regularly but quit, currently smoke occasionally, and currently smoke regularly. As only one participant had a history of smoking, smoking status was dichotomized into never and former smoker.

Health factors included frequency of health services utilization (a few times a year, 1–3 times a month, once a week, and at least twice a week) and self-rated health (very good, good, fair, bad, and very bad). None of the demographic, lifestyle or health variables had missing values.

Women were asked if they had heard of cervical cancer, if they knew the name of at least one cervical cancer screening test (HPV testing, Pap smear/test, VIA), and if they had had a test in the past five years. Participants were also asked to select from a list what they know about cervical cancer, with a write-in option presented for “other”.

Reason for not having had a screening test (HPV testing, Pap smear/test, VIA) was collected as a categorical variable, with the following options: not aware of the test, not convenient, fear, asymptomatic so no test is need, feeling embarrassed, feeling discomfort/pain, not confident about test result, test is too costly, no time, with a write-in other option. Only participants who had heard of a given test were asked if they had undergone that test and, if applicable, their reason for not getting tested.

There were minimal missing observations (<5) for knowing the name of at least one cervical cancer screening test.

All 164 participants provided a self-collected sample. Following self-collection, they were asked about the self-collection process: would they use the HPV test again in the future, and if not, why, experience with the self-collection process, where they would prefer to collect the sample (self-collected at home, self-collected at the clinic or hospital, or provider-collected at the clinic or hospital). Participants were also asked how they would prefer to get their test results (by phone, by post, or at the clinic), if they would be willing to return for a follow-up visit if they tested positive, and whether financial concerns play a role in their decisions about testing.

Acceptability of HPV self-sampling was measured through a binary (y/n) variable measuring whether participants would be willing to use the self-collection test again. Embarrassment, discomfort/pain, convenience, confidence doing the test, and financial concerns were evaluated on 5-point Likert scales. For purposes of this study, we defined feasibility as high acceptability of HPV self-sampling within this population, along with high confidence and convenience scores and low embarrassment, discomfort/pain, and financial concern scores. Knowledge of HPV risk factors and cervical cancer risk factors was evaluated via questions about HPV risk factors and cervical cancer risk factors. These measures were drawn from previous studies related to self-collection [29].

### Statistical analysis

The sociodemographic profile of the study population, knowledge of cervical cancer and screening, and the HPV self-sampling results, knowledge, and acceptability were described using counts and percentages by age group. The Kruskal-Wallis test was used to evaluate differences in all variables across age groups (30–39, 40–49, 50–65). Statistical significance was defined as p<0.05. All analyses were performed in R statistical software version 4.3.2.

### Inclusivity in global

Additional information regarding the ethical, cultural, and scientific considerations specific to inclusivity in global research is included in the S1 Checklist.

## Results

A total of 164 women were enrolled into the study (See Table 1). The mean age was 44.3 (standard deviation: 9.1), with ages ranging from 30–65 as per the inclusion criteria. Nearly all participants were married (88.4%). There were marked differences by age group in education attainment, with younger participants (30–39 years old) showing more years of schooling obtained especially at the higher secondary school and graduate educational levels compared to the oldest participants (50–65 years old; Table 1). Similarly, while most of the women reported being housewives (81.1%), participants in the 30–39 age group reported higher levels of employment than those in the older age groups. Differences in education and occupation across the age groups were statistically significantly different from each other (p-values = <0.05; Table 1).

**Table 1:** Cohort demographics by age (N = 164).

	30–39 (N = 53)		40–49 (N = 58)		50–65 (N = 53)	
	N (%)		N (%)		N (%)	
**Marital Status**						
Married	50	(94.3)	54	(93.1)	41	(77.4)
Divorced or separated	1	(1.9)	0	(0)	0	(0)
Widowed	2	(3.4)	4	(6.9)	12	(22.6)
Education*						
None (0 years of schooling)	3	(5.7)	10	(17.2)	22	(41.5)
Primary school (1–5 years)	13	(24.5)	10	(17.2)	18	(34.0)
Secondary school (6–10 years)	17	(32.1)	24	(41.4)	12	(22.6)
Higher secondary school (11–12 years)	8	(15.1)	4	(6.9)	1	(1.9)
Graduate (13–16 years)	7	(13.2)	3	(5.2)	0	(0)
Postgraduate (17 years)	5	(9.4)	7	(12.1)	0	(0)
Occupation*						
Factory worker	2	(3.8)	2	(3.5)	0	(0)
Service worker	15	(28.3)	9	(15.5)	3	(5.7)
Housewife	36	(67.9	47	(81.0)	50	(94.3)
**Smoking status**						
Never smoker	53	(100.0)	58	(100.0)	52	(98.1)
Former smoker	0	(0)	0	(0)	1	(1.9)
Number of times pregnant*						
None	2	(3.8)	2	(3.5)	0	(0)
One	5	(9.4)	3	(5.2)	0	(0)
Two	18	(34.0)	11	(19.0)	2	(3.8)
Three	13	(24.5)	9	(15.5)	10	(18.9)
Four or more times	15	(28.3)	33	(56.9)	41	(77.4)
Number of living children*						
None	2	(3.8)	2	(3.5)	0	(0)
One	7	(13.2)	7	(12.1)	0	(0)
Two	24	(45.3)	14	(24.1)	7	(13.2)
Three	13	(24.5)	19	(32.8)	19	(35.9)
Four or more	7	(13.2)	16	(27.6)	27	(50.9)
Frequency of health service utilization*						
A few times a year	22	(41.5)	11	(19.0)	7	(13.2)
1–3 times a month	28	(52.8)	45	(77.6)	37	(69.8)
Once a week	3	(5.7)	2	(3.5)	9	(17.0)
At least twice a week	0	(0)	0	(0)	0	(0)
Self-rated health*						
Very good	0	(0)	1	(1.7)	1	(1.9)
Good	20	(37.7)	14	(24.1)	5	(9.4)
Fair	22	(41.5)	18	(31.0)	19	(35.9)
Bad	11	(20.8)	24	(41.4)	28	(52.8)
Very bad	0	(0)	1	(1.7)	0	(0)

* denotes significance at p = <0.05

Only one participant reported a history of smoking. There were differences by age group in gravidity, with the majority of the older age groups having had four or more pregnancies. Comparatively, the majority of people in the 30–39 age group had been pregnant either two or three times, with 34% reporting having had two pregnancies. A majority of the oldest age group had four or more living children; in contrast, 45.3% of the people in the 30–39 age group had two living children. Number of times pregnant, and number of living children were found to be statistically significantly different (p-values = <0.05; Table 1).

Health services utilization among the cohort was high, with 67.1% reporting using health services between 1–3 times a month (Table 1). Use was highest amongst participants in the 50–65 age group. The majority of participants rated their health as “Bad” (38.4%) or “Fair” (36%). Older participants were more likely to rate their health as “bad” (52.8%) compared to those in the 40–49 age group and 30–39 age group (41.4% and 20.8%, respectively). Participants in the 30–39 age group were more likely to rate their health as “good” than the older age groups. Self-rated health, and frequency of health service utilization were statistically significantly different across age groups (p-values = <0.05; Table 1).

Only three participants had not heard of cervical cancer; 95.7% (95% CI: 91.3–98.2%) of those who had heard of cervical cancer knew that cervical cancer is a form of cancer that affects the lower genital tract of women. Less than 10% of participants in both the 30–39 (95% CI: 1.1–15.7%) and 40–49 (5.2%, 95% CI: 1.1–14.4%) age groups and no participants in the oldest age group, 50–65 knew that HPV is a sexually transmitted infection (95% CI: 0.0–6.7%). There were differences in knowing the name of at least one cervical cancer screening test by age group: twice the number of those in the 30–39 age group knew of a cervical cancer screening test by name compared to those in the 50–65 age group. Every participant who knew the name of a screening test (n = 45) had heard of VIA. Very few participants (N = 7) had heard of a Pap test, none of whom were in the 50–65 age group. Knowing the name of one cervical cancer screening test and having heard of VIA were found to be statistically significantly different between the groups (p-values = <0.05; Table 2).

**Table 2:** Knowledge of cervical cancer and screening by age group.

	30–39 (N = 53)		40–49 (N = 58)		50–65 (N = 53)	
	N (% - 95% CI)		N (% - 95% CI)		N (% - 95% CI)	
**Heard of cervical cancer**	52	(98.1)	57	(98.3)	52	(98.1)
**Knows cervical cancer is a form of cancer that affects the lower genital tract of women**	51	(96.2)	54	(93.1)	49	(92.5)
Know the name of at least one cervical cancer screening test **	40	(75.5)	34	(58.6)	20	(37.7)
**Knows that HPV is a sexually transmitted infection**	2	(3.8)	2	(3.5)	0	(0)
**Heard of Pap test**	3	(5.7)	4	(6.9)	0	(0)
Heard of VIA test**	40	(75.5)	34	(58.6)	20	(37.7)
**Had VIA test in past 5 years**	18	(34.0)	14	(24.1)	13	(24.5)
Reason for not having a VIA test (among those who had not had a test in the past 5 years)*						
Not aware of the test	12	(34.3)	24	(54.6)	32	(80.0)
Fear	3	(8.6)	2	(4.6)	1	(2.5)
Asymptomatic/no need for testing	21	(60.0)	18	(40.9)	3	(7.5)
Feeling embarrassed	2	(5.7)	2	(4.6)	0	(0)

* will sum to more than those who have not had a VIA test as participants were able to select more than one reason

** denotes significance at p = <0.05

Uptake of screening was low, with only 27.4% (95% CI: 20.8–34.9%) having been screened by VIA in the past five years. The youngest age group had the highest rates of VIA screening at 34% (95% CI: 21.5–48.3%). Most participants who had not been screened by VIA indicated that not being aware of VIA testing was the reason why they had not been screened, with the 50–65 age group being least likely to have been aware of the test. Among those aged 30–39, the primary reason for not being screened was being asymptomatic and therefore believing there was no need for testing. Few participants cited feelings of fear and embarrassment as barriers to screening (Table 2).

High-risk HPV positivity was 4.3% overall (n = 7, 95% CI: 1.7–8.6%), but in the 30–39 age group was 5.7% (1.2–15. 7%) compared to 3.5% (95% CI: 0.4–11.9%) and 3.8% (95% CI: 0.5–13.0%) in the 40–49 and 50–65 age groups, respectively. However, there was no statistically significant difference in positivity by age group. A total of 12 tests (7.4%, 95% CI: 3.4–12.4%) were borderline (Table 3). Concordance between the self-collected and provider-collected samples was 93.3%; two samples were borderline by self-collection and negative by provider-collection.

**Table 3:** HPV self-sampling results, knowledge, and acceptability by age group.

	30–39 (N = 53)		40–49 (N = 58)		50–65 (N = 53)	
	N (%)		N (%)		N (%)	
**HPV self-sampling results**						
Positive	3	(5.7)	2	(3.4)	2	(3.8)
Negative	45	(84.9)	51	(87.9)	49	(92.5)
Borderline	5	(9.4)	5	(8.6)	2	(3.8)
**Would you use the HPV test again in the future?**						
Yes	53	(100)	56	(96.6)	48	(90.6)
No, asymptomatic, so no need	0	(0)	0	(0)	1	(1.9)
No, test is too costly	0	(0)	2	(3.4)	4	(7.5)
**Overall, how do you feel about this sample collecting process for HPV test?**						
Very good	8	(15.1)	7	(12.1)	6	(11.3)
Good	42	(79.3)	47	(81)	42	(79.3)
Fair	3	(5.7)	4	(6.9)	5	(9.4)
Bad	0	(0)	0	(0)	0	(0)
Very bad	0	(0)	0	(0)	0	(0)
**How convenient do you think collecting this self-sample is?**						
Not convenient	0	(0)	0	(0)	0	(0)
A little convenient	0	(0)	0	(0)	0	(0)
Fair	3	(5.7)	4	(6.9)	5	(9.4)
Convenient	42	(79.2)	47	(81)	42	(79.2)
Very Convenient	8	(15.1)	7	(12.1)	6	(11.3)
**How embarrassed were you to collect this self-sample?**						
Not embarrassed/pain	51	(96.2)	52	(89.7)	47	(88.7)
A little embarrassed/pain	2	(3.8)	6	(10.3)	6	(11.3)
Fairly embarrassed	0	(0)	0	(0)	0	(0)
Embarrassed/pain	0	(0)	0	(0)	0	(0)
Very embarrassed	0	(0)	0	(0)	0	(0)
**How much discomfort/pain did you experience while collecting this self-sample?**						
No discomfort/pain	51	(96.2)	52	(89.7)	48	(90.6)
A little discomfort	1	(1.9)	5	(8.6)	5	(9.4)
Fair	1	(1.9)	1	(1.7)	0	(0)
Discomfort/pain	0	(0)	0	(0)	0	(0)
Severe discomfort/pain	0	(0)	0	(0)	0	(0)
How confident are you that you collected the sample correctly?*						
Very confident	14	(26.4)	11	(19)	3	(5.7)
Confident	38	(71.8)	43	(74.1)	44	(83)
Fair	1	(1.9)	4	(6.9)	5	(9.4)
A little confident	0	(0)	0	(0)	1	(1.9)
Not confident	0	(0)	0	(0)	0	(0)
**Did you experience any difficulties collecting this sample?**						
Yes, felt a little pain	1	(1.9)	3	(5.2)	5	(9.4)
No	52	(98.1)	55	(94.8)	48	(90.6)
**How would you prefer to collect this sample?**						
At home, by myself	9	(17)	9	(15.5)	4	(7.5)
At the clinic or hospital, by myself	44	(83)	47	(81)	46	(86.8)
At the clinic or hospital, by physician or nurse	0	(0)	2	(3.4)	3	(5.7)
**How would you prefer to get your test results?**						
By post	0	(0)	0	(0)	1	(1.9)
At the clinic	10	(18.9)	6	(10.3)	3	(5.7)
By telephone	43	(81.1)	52	(89.7)	49	(92.5)
**If the test is positive, would you be willing to come to the hospital for a follow-up test?**						
Yes	51	(96.2)	58	(100)	51	(96.2)
No, financial difficulties	2	(3.8)	0	(0)	2	(3.8)
						()
**Is money one of your concerns when conducting HPV test (∼2500 Bangladeshi Taka/23 USD)?**						()
No concern	40	(75.5)	42	(72.4)	38	(71.7)
A little concerned	8	(15.1)	9	(15.5)	4	(7.5)
Fair	0	(0)	2	(3.4)	0	(0)
Concerned	3	(5.7)	2	(3.4)	6	(11.3)
Very concerned	2	(3.8)	3	(5.2)	5	(9.4)

* denotes significance at p = <0.05

There was wide acceptability of the self-collection process, with 95.7% (95% CI: 91.4–98.3%) of all participants indicating that they would self-sample again in the future (Table 3). Among those who would not self-sample in the future, monetary concerns were the primary reason. No participants felt “very badly” or “badly” about sample collection process; generally, participants in all age groups felt “good” or “very good” about the self-collection process (92.7%, 95% CI: 87.9–96.2%).

The self-collection process was viewed as convenient, and embarrassment was low (Table 3). Participants in all age groups felt little discomfort, particularly in the youngest age group.

Confidence with respect to self-collection was rated as either “confident” or “very confident” by 98.1% (95% CI: 90–100%) of the 30–39 age group. The older age groups reported slightly lower levels of confidence: while 93.1% (95% CI: 83.3–98.1%) of the 40–49 age group rated themselves as “confident” or “very confident”, the oldest age group, 50–65, had the lowest levels of confidence. Confidence in ability to self-sample was statistically significantly different by age group (p-value = <0.05; Table 3).

Participants were also asked where they would be comfortable doing the self-collection, and a majority indicated that they would prefer to collect their sample by themselves at the clinic or hospital; only a small percentage of participants reported not wanting to self-collect (Table 3). Overall, most participants indicated that they would not be comfortable collecting their sample at their home (Table 3).

Almost every participant responded that they would come to the hospital for a follow-up test, except for 2.43% (95% CI: 0.7–6.1%) who cited financial difficulties as the barrier to their return for a follow-up. Similarly, 12.8% (95% CI: 8.1–18.9%) of participants reported being “very concerned” or “concerned” about money with respect to the HPV test, which costs approximately 2,500 Bangladeshi Taka, or 23 USD (Table 3).

## Discussion

This is the first study in Bangladesh to assess HPV self-sampling feasibility and acceptability and provides a snapshot of prevalence in this community situated outside of Dhaka. While nearly all participants had heard of cervical cancer, specific knowledge was quite low, which was reflected in screening rates. Reported past screening overall was slightly over 25%, with about one-third of women in the 30–34-year-old group reporting ever being screened.

The cervical cancer control program in Bangladesh consists of a population-based VIA screening program. Every married woman over 30 is offered screening using VIA and referred to colposcopy and treatment if found positive [23]. Previous reports suggest that less than 10% of the population is screened currently [25], but our results suggest this may be higher. However, given the presence of a health clinic in the community, this may be artificially high relative to the rest of the country.

High-risk HPV positivity was 4.3% overall with higher rates observed in the 30–39 age group compared to the 40–49 and 50–65 age groups. A total of 12 tests were borderline, which could indicate the sample was not collected properly or that the level of detectable HPV fell short of appearing positive. However, the high levels of concordance of self-collected samples with physician collected samples demonstrate its ability to return accurate results. With high rates of acceptability across different age groups, high rates of convenience, and very little discomfort and embarrassment reported, HPV self-sampling as a form of cervical cancer screening in Bangladesh appears feasible.

HPV self-sampling has been introduced as a primary method of cervical cancer screening or as a secondary method, used to reach those who are under screened. While some cancer program programs mail kits to patients, others provide them at local health clinics or through local health workers [30]. HPV self-sampling has proven to be an effective component of cervical cancer control programs due to its ability to be used at home or at local health clinics [30]. Bangladesh can implement HPV self-sampling by offering it at all primary health facilities, at the community, union-, and Upazila-level [31]. As self-sampling is not as labor- or resource-intensive as traditional forms of screening, it fits into Bangladesh’s efforts to advance Universal Health Coverage [24, 31].

However, there are several limitations to this study: not all relevant risk factor information was collected, due to cultural sensitivities. This prevented us from being able to evaluate if there were any differences in HPV prevalence or self-sampling acceptability by risk factor profiles. Additionally, participants were drawn from attendees of a local clinic, which could limit the generalizability of the results. We did not include questions about the usefulness of the instructions and therefore we can not evaluate the their impact on the self-collection process. Due to our sample size, the precision around HPV prevalence in this population is not high. Lastly, we lack follow-up data regarding those with positive tests and thus do not know if any follow-up was sought, and if any cervical cancers were detected among the screen-positive participants.

Next steps include replicating and expanding this study beyond attendees of this clinic in the Araihazar Upazila in Bangladesh. Future studies should look at various localities and identify participants through other health care centers, community centers, radio and print advertising, and word of mouth, and potentially through door-to-door recruitment initiated by community health workers. Furthermore, to ascertain feasibility of implementation as a part of a national cancer control program, a cost-effectiveness study should be undertaken to understand the financial implications on both government and patients.

## Supporting information

S1 Checklist Inclusivity in global research. (DOCX)
